# WeiTsing: a new face of Ca^2+^-permeable channels in plant immunity

**DOI:** 10.1007/s44154-023-00110-4

**Published:** 2023-07-22

**Authors:** Feng-Zhu Wang, Jian-Feng Li

**Affiliations:** State Key Laboratory of Biocontrol, Guangdong Provincial Key Laboratory of Plant Resources, School of Life Sciences, Sun Yat-Sen University, Guangzhou, 510275 China

**Keywords:** Plant immunity, Ca^2+^-permeable channel, Endoplasmic reticulum, *Plasmodiophora brassicae*, *Brassica* crop

## Abstract

Plants employ pattern- and effector-triggered immunity (PTI and ETI) to synergistically defend invading pathogens and insect herbivores. Both PTI and ETI can induce cytosolic Ca^2+^ spikes, despite in different spatiotemporal patterns, to activate downstream Ca^2+^-dependent immune signaling cascades. While multiple families of Ca^2+^-permeable channels at the plasma membrane have been uncovered, the counterparts responsible for Ca^2+^ release from intracellular stores remain poorly understood. In a groundbreaking paper published recently by *Cell*, the authors reported that WeiTsing, an Arabidopsis endoplasmic reticulum (ER)-resident protein that was specifically expressed in the pericycle upon *Plasmodiophora brassicae* (*Pb*) infection, could form resistosome-like Ca^2+^-conducting channel and protect the stele of *Brassica* crops from *Pb* colonization. As the channel activity of WeiTsing was indispensable for its immune function, the findings highlight a previously underappreciated role of Ca^2+^ release from intracellular repertoire in promoting plant disease resistance.

## Main text

Lacking an adaptive immune system, plants solely rely on the innate immune system to counteract pathogen infection and herbivore infestation. Plant innate immunity can be conceptually categorized into pattern-triggered immunity (PTI) and effector-triggered immunity (ETI) (Zhou and Zhang 2020). PTI is activated by plasma membrane (PM)-localized immune receptors, termed pattern-recognition receptors (PRRs), through perceiving so-called microbe/herbivore-associated molecular patterns (MAMPs/HAMPs) or endogenous damage-associated molecular patterns (DAMPs), which confers basal and broad-spectrum resistance. ETI is initiated by intracellular nucleotide-binding leucine-rich repeat receptors (NLRs) upon detecting invader-delivered virulence factors (effectors) or consequent perturbation effects on PTI, leading to intense but race-specific resistance. PTI and ETI share many overlapping immune responses, such as cellular Ca^2+^ influx, and can form mutually potentiated immune circuits. Meanwhile, locally occurring PTI or ETI can induce the production of secondary mobile molecules that travel to distal tissues to trigger systemic acquired resistance (SAR) against subsequent attacks.

Cytosolic Ca^2+^ serves as an essential second messenger in eukaryotic cells. In resting plant cells, cytosolic Ca^2+^ must be maintained at low levels (~ 100 nM) to avoid forming insoluble and cytotoxic Ca_3_(PO_4_)_2_ with abundant phosphate ions, while free Ca^2+^ at much higher concentrations is sequestered in the apoplast (~ 10 mM) and intracellular organelles, such as vacuoles (up to 5 mM) and endoplasmic reticulum (ER, ~ 5 μM) (Cortese et al. 2022). During PTI or ETI activation, cytosolic Ca^2+^ spike represents one of the hallmark immune responses. The spatiotemporal dynamics of cytosolic Ca^2+^ elevation (also known as Ca^2+^ signatures) are subsequently deciphered by various types of Ca^2+^ decoders, such as calmodulins (CaMs), Ca^2+^-dependent protein kinases (CDPKs) or metacaspases (Shen et al. 2019), which in turn modulate diverse cellular processes to amplify immunity or provide feedback regulation.

**Fig. 1:**
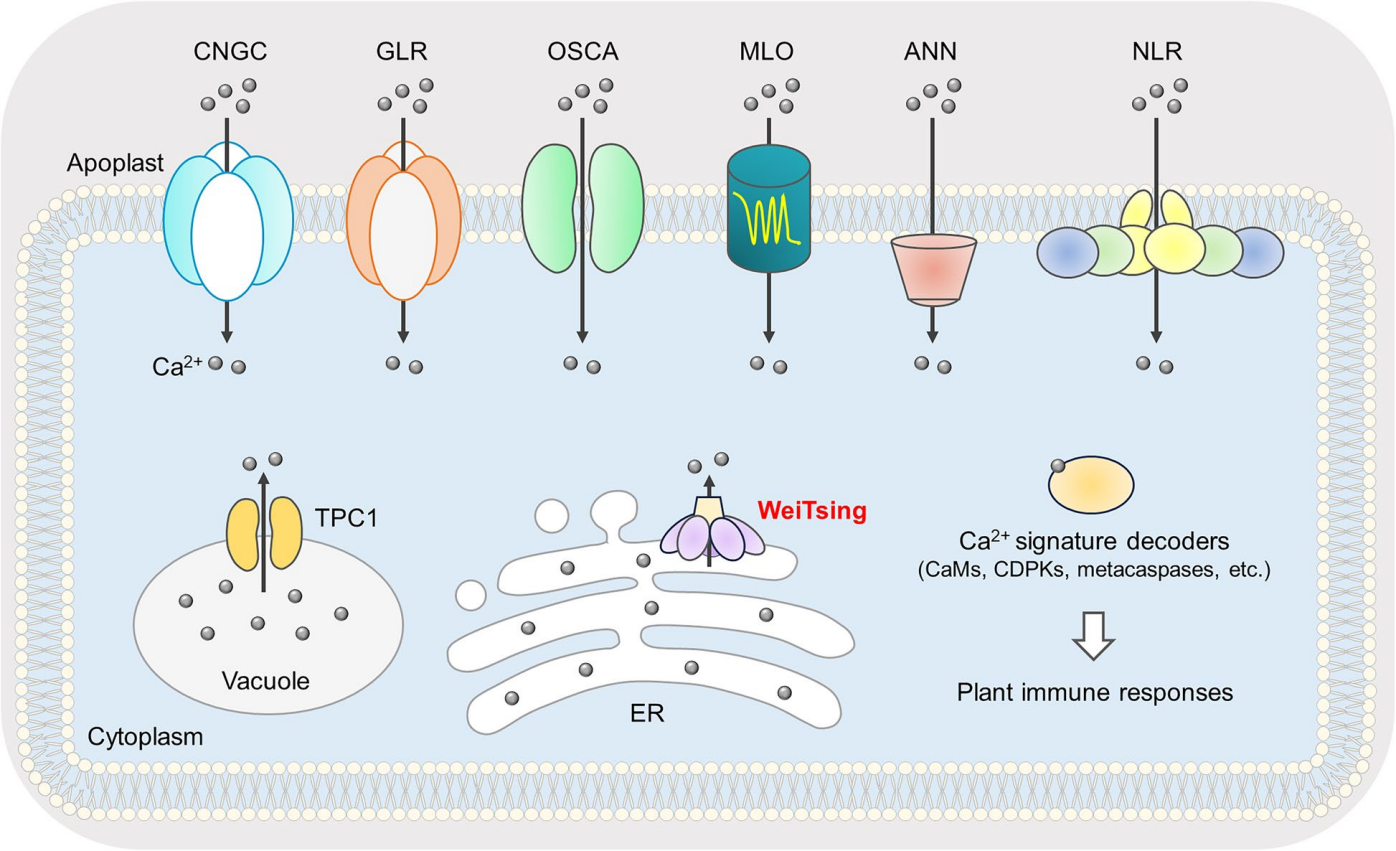
Multiple families of Ca^2+^-permeable channels in plant immunity. Plasma membrane-localized canonical Ca^2+^-permeable channels, including CNGCs, GLRs, and OSCAs, and non-canonical Ca^2+^ channels, including ANNs, MLOs, and NLR complexes (resistosomes), can mediate Ca^2+^ influx from the apoplast upon PTI or ETI activation. During plant immunity, tonoplast-resident Ca^2+^-conducting channel TPC1 and the newly discovered ER-resident Ca^2+^-conducting channel WeiTsing (highlighted in red) can mediate Ca^2+^ release from the vacuole and ER, respectively. Distinct spatiotemporal dynamics of cytosolic Ca^2+^ elevation (also known as Ca^2+^ signatures) are shaped by coordinated action of different plasma membrane and organellar calcium channels in a cell type-specific manner. In turn, Ca^2+^ signatures are decoded by various Ca^2+^ sensors to regulate downstream immune responses

Precisely shaped Ca^2+^ signatures in plants in response to distinct immune signals require coordinated action of Ca^2+^-permeable channels at the PM and organellar membranes, which control Ca^2+^ influx from the apoplast and Ca^2+^ release from the intracellular stores, respectively. So far, multiple families of canonical or noncanonical Ca^2+^-permeable channels at the PM have been reported in Arabidopsis (Xu et al. 2022; Köster et al. 2022), including CYCLIC NUCLEOTIDE-GATED CHANNELs (CNGCs), GLUTAMATE RECEPTOR-LIKEs (GLRs), REDUCED HYPEROSMOLALITY-INDUCED [Ca^2+^] INCREASEs (OSCAs), ANNEXINs (ANNs), MILDEW RESISTANCE LOCUS Os (MLOs), and the ion channels formed by coiled-coil (CC) or helper NLR complexes (Fig. 1). For example, CNGC2 and CNGC4 can form hetero-tetramer channels to mediate the bacterial MAMP flg22-induced Ca^2+^ influx (Tian et al. 2019), while CNGC6 and CNGC19 are engaged in the Ca^2+^ influx induced by the DAMPs eATP (Duong et al. 2022) and Pep1 (Meena et al. 2019), respectively. GLR3.3 and GLR3.6 are able to form oligomeric channels for propagating systemic Ca^2+^ signals from the herbivore feeding site to distal leaves through the vasculature (Toyota et al. 2018), while GLR2.7 and GLR2.9 are transcriptionally upregulated in response to multiple MAMPs/DAMPs to mediate Ca^2+^ influx (Bjornson et al. 2021). OSCA1.3 and OSCA1.7 act as homo-dimeric Ca^2+^ influx channels in response to flg22 exclusively in guard cells (Thor et al. 2020). ANN1, a cytosolic protein without transmembrane spans, participates in the fungal MAMP chitin-induced Ca^2+^ influx (Espinoza et al. 2017) as well as herbivory-induced local and systemic Ca^2+^ signaling (Malabarba et al. 2021). MLO2, a seven-transmembrane protein rendering plant susceptibility to powdery mildew fungi, has been shown as a Ca^2+^-permeable channel (Gao et al. 2022). Strikingly, upon detecting the bacterial effector AvrAC, the CC-NLR ZAR1 (HOPZ-ACTIVATED RESISTANCE 1) is able to form a pentameric complex, termed resistosome, which subsequently creates a Ca^2+^-permeable pore at the PM (Bi et al. 2021). Similarly, the helper NLRs NRG1 (N REQUIREMENT GENE 1) and ADR1 (ACTIVATED DISEASE RESISTANCE 1), when activated by the toll/interleukin-related (TIR)-NLRs upon effector recognition, are capable of forming oligomeric complexes at the PM to confer Ca^2+^ permeability (Jacob et al. 2021). The MIXED LINEAGE KINASE DOMAIN-LIKE (MLKL) proteins, which also function downstream of TIR-NLRs and exhibit partial structural similarity to NRG1 and ADR1, are able to form tetrameric complex that is postulated to generate a pore-like structure at the PM via the HeLo domain (Mahdi et al. 2020). Unlike PM-resident Ca^2+^-conducting channels, their intracellular counterparts have remained largely unknown except TPC1 (TWO-PORE CHANNEL 1), which can form a dimeric channel on the tonoplast (Fig. 1) and is involved in herbivory-induced local and systemic Ca^2+^ signaling (Vincent et al. 2017).

Recently, a remarkable work by Wang and colleagues has discovered for the first time an ER-localized Ca^2+^-permeable channel (Fig. 1) that can confer *Brassica* crops with broad-spectrum resistance to the devastating clubroot disease caused by the soil-borne pathogen *Plasmodiophora brassicae* (*Pb*) (Wang et al. 2023). In the study, the authors started with screening 117 Arabidopsis natural accessions for enhanced *Pb* resistance, which allowed them to identify the Est-1 accession that was highly resistant to 16 *Pb* isolates collected throughout China. Through genetic analysis of crossing progenies between Est-1 and Col-0, a *Pb* susceptible accession, the resistance gene was mapped to a ~ 45-kb region on chromosome 1 in Est-1, which was absent in Col-0. The authors then focused on four tandemly arrayed genes (i.e., C6 to C9) in this region, among which only *C6* could lead to an autoimmune phenotype in Col-0 when expressed by a 2-kb native promoter, whereas *C6* knockout in Est-1 caused *Pb* susceptibility. Of particular note, when *C6* (later renamed *WeiTsing*) was expressed by a 3.6-kb native promoter (*p3.6k*) in Col-0, the transgenic plants appeared to grow normally while retaining *Pb* resistance. Transgenic introduction of *p3.6k-WeiTsing* into the oilseed rape (*Brassica napus*) also conferred broad-spectrum *Pb* resistance without growth penalty. These results suggest that *WeiTsing* is the clubroot resistance gene and its activity is tightly modulated by transcriptional regulation. Consistently, the authors found that *WeiTsing* was expressed at low levels in un-inoculated Est-1 roots, but was massively induced by inoculation with all 16 *Pb* isolates. Interestingly, the promoter of *WeiTsing* in the L*er* accession was defected. Accordingly, *WeiTsing* in L*er* could not be induced by *Pb* infection to confer resistance, whereas transgenic introduction of the *p3.6k* promoter from Est-1 in combination with the *WeiTsing* coding sequence from L*er* could establish *Pb* resistance in Col-0, suggesting that the *p3.6k* promoter is key for *Pb* resistance. Noteworthily, a preprint paper also reported the same gene (named *RPB1*) as a *Pb* resistance gene (Ochoa et al. 2022). However, based on the observation that *RPB1* expression under a 1-kb native promoter was unable to confer full *Pb* resistance in Col-0, Ochoa and co-workers thought that *RPB1* is insufficient for conferring clubroot resistance. That study again reflects the importance of an intact promoter of *WeiTsing* for conferring *Pb* resistance.

Next, Wang and colleagues examined the tissue specificity and inducibility of the *p3.6k* promoter. Intriguingly, *WeiTsing* was exclusively expressed in the pericycle, where it could be induced by *Pb* as early as 11 days post inoculation. In line with the observation, *WeiTsing* knockout in Est-1 only impaired *Pb* resistance in the stele at late infection stages (i.e., 17 days post inoculation), suggesting that *WeiTsing* specifically functions in the pericycle to protect the stele from *Pb* colonization.

Then, how does *WeiTsing* mediate *Pb* resistance in the pericycle? To answer this question, the authors looked into *WeiTsing*-dependent transcriptional changes in Arabidopsis and oilseed rape upon *Pb* inoculation and found that the *Pb*-induced *WeiTsing* expression led to transcriptional activation of many defense-related genes. Moreover, the estradiol-induced *WeiTsing* expression was sufficient to trigger immune responses in the absence of *Pb* infection. Notably, WeiTsing was localized to the ER and could form oligomers in both co-immunoprecipitation and gel infiltration assays. The oligomeric structure of WeiTsing was subsequently solved by cryo-electron microscopy, which exhibited a symmetrical pentameric architecture with a central pore, reminiscent of the CC-NLR-containing resistosome. By using multiple electrophysiological approaches, the authors verified the pentameric WeiTsing complex as a cation-selective channel permeable to Ca^2+^. Taking advantage of the Ca^2+^ sensor GCaMP6m, they provided *in planta* evidence that WeiTsing is a Ca^2+^-permeable channel. Structure-guided mutagenesis further indicated that WeiTsing-mediated immune responses require its channel activity.

In summary, Wang et al. (2023) elegantly demonstrated a novel plant defense mechanism against a root pathogen, where the infection of *Pb* stimulates the expression and activity of an ER-resident Ca^2+^-conducting channel in pericycle cells, leading to fortified pericycle immunity to safeguard the stele. This study not only greatly improves our understanding about the intracellular Ca^2+^ channels controlling organellar Ca^2+^ release but also opens up a new avenue for genetic engineering of clubroot resistance in *Brassica* crops. Future studies are needed to determine how *Pb* induces *WeiTsing* expression in Est-1and how WeiTsing coordinates with PM-localized Ca^2+^ channels to regulate *Pb*-induced Ca^2+^ signature. The mechanism for reprograming the expression of defense-related genes by WeiTsing-mediated Ca^2+^ signaling in the pericycle upon *Pb* infection also remains to be characterized. Furthermore, because many soil-borne pathogens can breach the stele to cause diseases, it is tempting to speculate that WeiTsing also positively regulates the pericycle immunity against some of those pathogens.

## Data Availability

Not applicable.

## Abbreviations

PTI: Pattern-triggered immunity
ETI: Effector-triggered immunity
ER: Endoplasmic reticulum
*Pb*: *Plasmodiophora brassicae*
PM: Plasma membrane
PRR: Pattern-recognition receptor
MAMP: Microbe-associated molecular pattern
HAMP: Herbivore-associated molecular pattern
DAMP: Damage-associated molecular pattern
NLR: Nucleotide-binding leucine-rich repeat receptor
SAR: Systemic acquired resistance
CaM: Calmodulin
CDPK: Ca^2+^-dependent protein kinase
CC: Coiled-coil
TIR: Toll/interleukin-related
